# Selfish Behavior in IEEE 802.11ah Networks: A Detection Algorithm and Mitigation Strategies

**DOI:** 10.3390/s22124472

**Published:** 2022-06-13

**Authors:** Yuliyan Georgiev, Richard Verhoeven, Nirvana Meratnia

**Affiliations:** Department of Mathematics and Computer Science, Eindhoven University of Technology, 5600 MB Eindhoven, The Netherlands; p.h.f.m.verhoeven@tue.nl (R.V.); n.meratnia@tue.nl (N.M.)

**Keywords:** IEEE 802.11ah, WiFi HaLow, Internet of Things, RAW, selfish behavior, fairness, mitigation, detection, award and punishment, throughput

## Abstract

One of the latest protocols developed for the Internet of Things networks is IEEE 802.11ah, proposed by the WiFi Alliance. The new channel access mechanism in IEEE 802.11ah, which is called Restricted Access Window, aims at reducing the contention between the stations by allowing only selected stations to transmit data at certain time slots. Stations may exhibit selfish behavior to maximize their own throughput. This will come at the cost of the overall network quality of service. In this paper, we first analyze the default behavior of the IEEE 802.11ah protocol in terms of fairness. We then introduce various percentages of selfish stations and observe how the network’s quality of service in terms of fairness, throughput and packet-loss are affected. After establishing the inherent fairness of IEEE 802.11ah, we analyze applicability of two existing selfish behavior detection algorithms designed for IEEE 802.11 to the IEEE 802.11ah protocol. Due to their poor performance, we propose a new definition of ’selfish behavior’ specifically for IEEE 802.11ah, based on which we present a new algorithm for detecting selfish behavior. To combat selfish behavior and to create a better fairness, throughput and lower packet loss, we consequently present a novel mitigation algorithm called Selfish Stations Quarantine Punishment Algorithm (SSQPA). The proposed algorithm takes advantage of the RAW grouping to isolate selfish stations from the honest stations, thus mitigating the effect of the selfish behavior. SSQPA comes in two variants: honest stations-centric and network-centric. Our experimental results show that both variants can successfully mitigate selfish behavior effects in IEEE 802.11ah networks and either one can be used depending on the goal of the network.

## 1. Introduction

Many industries are adopting Internet of Things (IoT) technology to automate different processes, to avoid manual labour, to perform predictive maintenance, or to speed up development. Recent trends show no sign of slowing down the process of utilizing IoT technology. The goal of IoT is to connect billions of constrained devices to the Internet. As the number of devices participating in the network increases, their competition to access the communication channel and consequently the channel contention increases as well. This may result in data loss.

One of the latest WiFi protocols published by the WiFi Alliance primarily for IoT networks is IEEE 802.11ah, which is also known as WiFi HaLow. It has multiple novel features compared to the legacy IEEE 802.11 protocol. WiFi Certified 6 provides improved power saving to support IoT capabilities in some applications; however, it lacks range and penetration. WiFi 4 offers high data rates for applications such as video streaming and internet surfing at the cost of high battery consumption. Therefore, the WiFi Alliance has introduced the IEEE 802.11ah for the IoT market [1].

IEEE 802.11ah operates in the sub-1 GHz frequency spectrum band, which allows a communication range of up to 1 km with high obstacle penetration [2]. The association identifier feature allows a single access point (AP) to be associated with 8192 stations, which aims at high scalability [3].

### 1.1. Restricted Access Window in IEEE802.11ah

**Figure 1:**
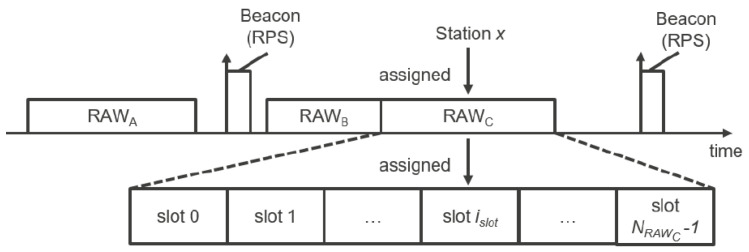
RAW assignment taken from [3].

Contention between stations in IEEE 802.11ah can be reduced using Restricted Access Windows (RAW), which provides a deterministic and stochastic medium access control mechanism as stated in [3]. As the name suggests, RAW provides a time window, during which channel access is restricted to certain stations only. During RAW, stations use an Enhanced Distributed Channel Access/Distributed Coordination Function (EDCA/DCF) to access the channel [3]. DCF uses Carrier-Sense Multiple Access with Collision Avoidance (CSMA/CA) with Binary Exponential Backoff (BEB) algorithm. With EDCA, traffic is prioritized depending on the traffic in four different access categories [4]. Information about the RAW organization is forwarded by AP using the RAW Parameter Set (RPS). The RPS is communicated during each beacon transmission by the AP and can be dynamically adjusted. Each RAW group is split into RAW slots and stations of a RAW group are evenly distributed over the RAW slots using Round Robin [5]. The duration of a slot is determined using Equation (1) [5]. The minimal duration of a slot is 500 μs when NRawSlotCount is equal to 0; the maximal duration is 246.14 ms when slot format is set to 1 and 31.1 ms when slot format is set to 0. All slots within a group have the same duration. A visual representation of the RAW assignment can be found in Figure 1. Stations can compete for medium access only during their RAW slot duration. Cross-Slot Boundary (CSB) is a setting that allows stations to continue an ongoing transmissions even if the RAW slot duration is exceeded if enabled. RAW can be restricted to paged traffic indication map stations only. In this case, only the paged stations will compete for the medium access in each RAW slot. Otherwise, all stations can compete.

(1) $$T_{slot}=500\;\mu s+NRawSlotCount\times 120\;\mu s$$

### 1.2. The Problem of Selfish Behavior in IEEE802.11ah

The selfish behavior of stations is a serious threat to the network’s quality of service in any wireless network, especially if there is no mitigation mechanism to deal with it. This is a problem that can occur in any type of network and has been greatly researched in IEEE 802.11 networks. The selfish behavior of a station can occur in different ways, which according to [6] are as follows:

- Scramble Clear To Send (CTS)—upon hearing Request To Send (RTS) from one station to the access point, the selfish station sends a message that will collide with the CTS response, causing the sender to backoff;
- Scramble Acknowledge (ACK) and DATA frames—same as above but for ACK or DATA frames;
- Reduced backoff time—stations draw the random backoff value from a set with smaller minimal value;
- Transmit before DCF InterFrame Spacing (DIFS)—stations begin transmission before the minimum time to transmit after the channel is idle has passed;
- Increase Network Allocation Vector (NAV) value in RTS or DATA frames to prevent stations in range from competing during this time.

Reducing the backoff time is a widely used scheme by selfish stations. This type of selfish behavior is achieved in the form of modifying the set from which the random backoff value is drawn after the channel is sensed to be busy. In IEEE 802.11ah and IEEE 802.11, the Contention Window (CW) is determined using the BEB algorithm. When a station using CSMA senses that the channel is busy for the first time, it draws a random CW value from the set [0, CW_min_]. After CW idle time slots are detected, a transmission is attempted. If it fails, the upper bound of the set is doubled and the procedure is repeated. The upper bound can reach CW_max_, which is usually 1023 time slots. Upon a successful transmission, the upper bound is reset to CW_min_. However, selfish stations have their lower bound set to any value, which can even be lower than the default CW_min_. This means that selfish stations in the network wait less before accessing the channel again and therefore have a higher chance of accessing the channel.

The fact that selfish stations have a higher chance of accessing the channel and therefore higher throughput comes at the cost of degrading the overall network quality. Therefore, it is essential to understand how and to what extent selfish stations will affect network performance in IEEE802.11ah, how selfish nodes can be detected and how their behavior can be combated to optimize overall network quality of service. To this end, our contribution can be summarized as follows:

- A thorough analysis of the inherent fairness of the IEEE 802.11ah protocol;
- Investigating applicability of two existing selfish behavior detection algorithms, i.e., Principle-agent method [7] and Deviation detection [8] mechanism designed for IEEE 802.11, to the IEEE 802.11ah protocol;
- Proposing a new definition of ’selfish behavior’ specifically for IEEE 802.11ah as well as an algorithm to detect stations exhibiting selfish behavior;
- Proposing a mitigation algorithm called Selfish Stations Quarantine Punishment Algorithm (SSQPA) to combat selfish behavior by utilizing an award and punishment concept.

## 2. Related Work

Although selfish behavior in IEEE 802.11 has been widely researched [9,10], the detection and prevention of selfish behavior in IEEE 802.11ah have not received equal attention. As such, detection and mitigation solutions available for IEEE802.11 may not directly be applicable to IEEE 802.11ah. IEEE 802.11ah uses RAW, which is meant to reduce the contention between stations and impact of selfish stations under RAW is unknown. In this section, we first describe some of existing algorithms for detection and mitigation of selfish behavior in wireless networks and in Section 5, we will investigate applicability of two of these for the IEEE802.11ah protocol.

### 2.1. Studying Selfish Behavior

Authors of [9] explained different types of selfish attacks and countermeasures for each attack. They divide attacks into either direct or indirect. Direct attacks include lowering medium access parameters, traffic remapping, and disregarding reservations, while indirect attacks include increasing medium access parameters, reporting false congestion, route disruption, and selective forwarding. Experimental evaluations of the actual effect of selfish attacks in the networks were presented in [11,12]. The former assessed the effect of backoff behavior in commercial network cards, while the latter studied backoff misbehavior in mesh networks. In [13,14], two different models based on Markov chains to derive the throughput of the stations under different traffic loads were proposed. The first paper focused on EDCA-based networks while the second paper focused on DCF-based networks. Both models showed that they can accurately predict the performance of the network when selfish stations are present and can be used for developing solutions to deal with the selfish stations.

The use of game theory is popular in defining effective strategies in communication networks. The authors of [8] used the Nash equilibrium [15] to demonstrate that the throughput achieved with presence of selfish stations is not the optimal point of a game. Furthermore, they found the Pareto-optimal point of the game using static game model from Nash bargaining framework. In [16], a distributed algorithm was proposed that is based on stochastic game theory that forces selfish stations to cooperate. In [10], the authors used Pareto-optimal Nash equilibrium and considered cases of a single and multiple cheaters. Finally, authors of [17] showed a game model to interpret IEEE 802.11 DCF and presented a fairness game model using a Nash equilibrium backoff strategy.

### 2.2. Detection of Selfish Stations

The detection of selfish stations may be passive, active or hybrid [9]. Passive detection algorithms do not require additional features to detect selfish stations. They are continuously monitoring the channel to determine whether a station is being selfish. In [6], the backoff period of stations was collected from different samples and was compared to a threshold. In [18], the detection algorithm collected data from all successfully received packets for a certain period and counted the time slots between them. A test was performed to check whether CW was drawn from a uniform set by comparing the empirical Cumulative Distribution Function (CDF) with the expected CDF of the idle slots using the Kolmogorov–Smirnoff goodness of fit test [19]. In [20], the authors presented an algorithm in which each station was monitored for a certain period, during which the number of successful transmissions was counted. Then, a recursive function that subtracted the upper bound of expected successful transmissions from the observed transmissions was used to determine whether the station was selfish.

Active detecting algorithms require the implementation of additional mechanisms to detect selfish behavior. The algorithm presented in [7] was developed for ad hoc IEEE 802.11b networks. It was placed on top of the protocol and could easily be adapted to star topology networks. The principal station is a special station in the network that monitors traffic, while agents are all other stations of the network. The principal stations try to maximize the throughput of the entire network, while agents try to maximize their own throughput. In [21], a modification to the IEEE 802.11 protocol was made in such a way that the backoff time duration for a station was determined by the access point in the network and the station was expected to use the provided value. In [22], a novel backoff generation module is proposed, which binds each station with a publicly known backoff value.

### 2.3. Countermeasures to Selfish Behavior

According to [9], countermeasures for selfish behavior attacks may be classified in three categories, i.e., prohibit, mitigate, and incentivize.

Prohibiting the attacks is the best countermeasure, because it eliminates all chances for a station to become selfish. The authors in [23] described preventing unauthorized manipulations of routing messages in mesh networks. However, it does not solve the backoff manipulation problem.

The second countermeasure is mitigation, for which its main idea is to mitigate the impact of selfish stations on the network so that honest stations maintain their throughput. This can be achieved by isolating the selfish stations from the honest ones. In [24], a distributed algorithm was presented that forces selfish stations to cooperate and forward packets using game theory. In [16], a distributed algorithm based on stochastic game theory was used to force selfish station to cooperate in multi hop networks. Packets were sent and received as a Bernoulli process [25] and the number of punishments was adapted on the discount factor to force selfish stations to cooperate. In [26], a mechanism was developed, in which stations cooperated and cheating would have resulted in the inability to cooperate again. The incentivize category as a countermeasure aims to eliminate advantages of the misbehaving stations. It is the most commonly used countermeasure and there are three different ways performing thisl, i.e, punishment, threat, and credit-based communication [9].

The most effective approach is to punish selfish stations. This can be performed by de-authentication, blacklisting, or dropping packet [9]. In [27], the authors completely replaced the BEB algorithm with a Predictable Random Backoff (PRB) algorithm to prevent two types of backoff misbehavior, i.e., naive and smart. In the former, the attacker has no notion of the intrusion detection system, while in the latter, the attackers adapt their behavior to avoid detection. The algorithm forces stations to follow the honest strategy because any deviation will be immediately detected by the access point and packets of misbehaving stations would be dropped as a penalty. Papers such as [10,21] also proposed punishment mechanisms. The penalty for violating the rules of the protocol in [21] is forcing the selfish stations to wait longer before their next transmission. In [10], the authors developed a localized distribution protocol that guides the selfish stations into a Pareto-optimal Nash equilibrium. In using threats as countermeasures, selfish stations are threatened with punishment if their behavior persists. This approach has been shown to be most efficient for traffic remapping attacks [4]. Furthermore, stations suffering from selfish stations can send a dissatisfaction message to indicate that. In credit-based communication, stations obtain credits for forwarding messages and they use them to send messages. In [28], stations use credits for sending packets. The cost for each packet is determined by the transmission duration and packet size. Stations are periodically granted credits if they do not have any. Using this algorithm, selfish stations will quickly run out of credits and will leave the channel free to the honest stations. In [29,30], the authors use virtual currency. In the former paper, however, additional temper-resistant software and hardware are used to store the currency. The latter paper relies on a central authority for managing the currency.

### 2.4. Selfish Behavior in IEEE 802.11ah

To the best of authors’ knowledge, the only paper that has researched the selfish behavior in IEEE 802.11ah is [31]. The authors studied the network throughput in a scenario, in which the network is infested with different proportions of misbehaving stations under RAW allocation. The analysis was based on evolutionary game theory. The number of players in the game (stations) was finite and each player could choose from two possible strategies, i.e., being honest or selfish. The game was non-cooperative and each player strove to optimize its own throughput. Players choosing selfish strategy had CW_min_ set to two units of slot time, while honest players had CW_min_ set to 15 slot time. Each player had a payoff and utility function that were used for determining the benefits of choosing each strategy. Finally, all players were uniformly distributed over RAWs and the game was played multiple times.

A mitigation solution proposed specifically for IEEE 802.11ah can be found in [32]. However, the solution was proposed for the association phase, not for RAW communication, and it was a form of prohibiting selfish attacks. Instead of letting stations draw the random backoff value during the association phase, the access point drew a random number and sent it to all stations that send an association request. Upon receiving the random value, the stations used a function to calculate their backoff value using the random value provided by the access point and the last three digits of their MAC address, which were also random. This approach could be adapted to RAW by including the random value in the beacon sent at the start of each RAW or by letting the stations draw the random variable themselves and still use the function to calculate the backoff value.

## 3. Simulation Set Up

The simulator used for this paper is NS-3 [33] with the IEEE 8022.11ah extension developed in [34]. The parameters used in the simulation can be found in Table 1. We consider a network of *N* stations ∈ {30, 50, 100}, divided into *k* RAW slots ∈ {1, 2, 4}. The stations are competing for the channel access to a single AP. We consider three different network traffic scenarios, i.e., UDP, TCP and TCP IP camera, each of which having different traffic intensity. In all scenarios, the stations constantly have data packets to send to the AP. The three scenarios we consider are as follows:

- A UDP communication, in which stations generate UDP packets every 0.1 s and send a packet to the AP when they obtain access to the channel.
- A TCP communication, in which the stations have TCP packets every 0.2 s. Stations send packets to the AP to check whether there is a firmware update. Note that the down-link communication of the TCP firmware from the AP to the stations is disabled so that only the up-link communication is monitored.
- A TCP communication, in which the stations are IP cameras and compete for channel access to stream a video footage for a short period of time.

The stations of the network are either honest or selfish. Honest stations obey the MAC layer and use the default CW_min_ of 15 units of slot time. Selfish stations are modified and their CW_min_ value is set to two units of slot time.

## 4. Inherent Fairness of IEEE 802.11ah and Effect of Selfish Behavior

In this section, we analyze fairness of IEEE 802.11ah without introducing any selfish stations. To calculate fairness, we use the Jain’s fairness function [35], which can be found in Equation (2), where *x*_i_ is the number of data transmission of station *i* and *N* is the number of stations. The fairness is a value between 0 and 1, where a higher value indicates a better fairness. Value 1 equals to 100% fairness, which means all stations have the same channel access and transmit the same number of packets.

(2) $$Fairness=(\sum_{i=1}^{N}x_{i}{)}^{2}/N\times \left(\sum_{i=1}^{N}x_{i}^{2}\right)$$

**Figure 2:**
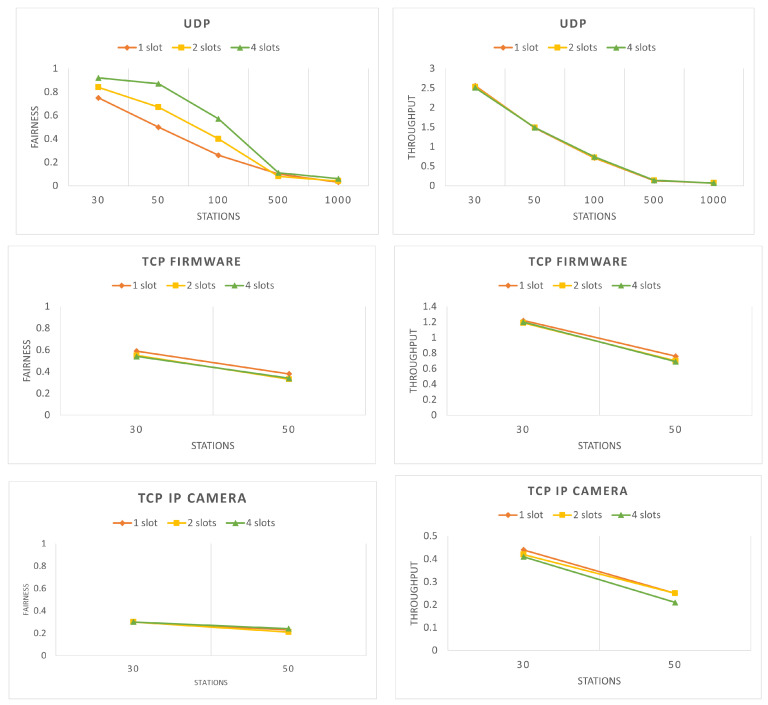
(**left**) Network fairness, (**right**) Network throughput (in Mbps)—in 60 s for different number of stations and RAW slots without introducing selfish stations.

As it can be seen in Figure 2 (left), the fairness of the network decreases with the increase in stations and increases with the increase in RAW slots. The fluctuations in the fairness are due to the fact that the larger the number of stations, the higher the contention. On the other hand, the higher the number of RAW slots, the lower the number of stations per slot; hence, contention decreases. However, if the network has more than 500 stations, it becomes very unfair and the difference between the three slot variations is not significant. Therefore, for the rest of our analysis, we only consider a maximum of 50 stations in the network. For the cases of 30 and 50 stations, the difference between the fairness of the three slot variations is not as noticeable for the TCP scenarios as for UDP. This is due to the different nature of the protocols. TCP is slower and, therefore, in general, there is less contention in the network between the stations than UDP and, hence, lower fairness variations.

The throughput for the different scenarios can be found in Figure 2 (right). It can be seen that throughput decreases with the increase in the number of stations, following a similar pattern to the one of the fairness. However, the number of RAW slots does not improve the throughput and only improves fairness. The different numbers of RAW slots result in almost the same throughput values; therefore, the lines of the graph are overlapping.

**Figure 3:**
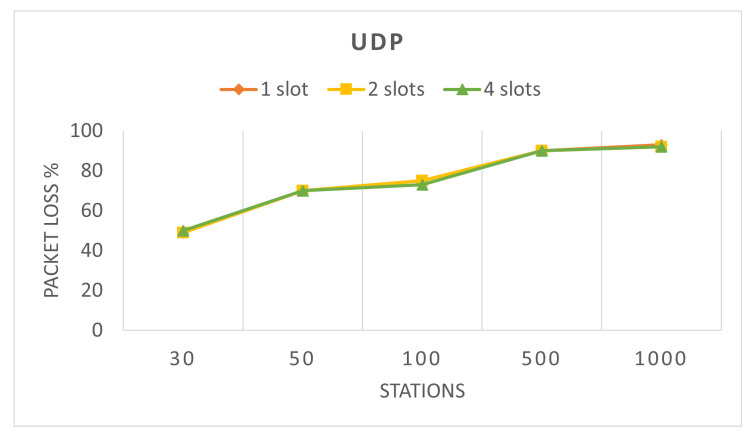
UDP network packet loss in 60 s for different number of stations and RAW slots without introducing selfish stations.

Finally, we analyze packet loss of the network. The source of packet loss in the network is only one and it is the limited transmission queue at the stations. Because the network always has packets to send, which are generated in every 0.1 s for UDP and 0.2 s for TCP, if a new packet is generated while the queue of the station is full, the packet will be dropped. Both TCP scenarios have 0 packet loss due to TCP’s higher reliability, lower data rate and lower packet generation interval; therefore, we do not illustrate them. As it can be seen in Figure 3, the packet loss for UDP is increasing with a higher number of stations. Furthermore, because packet loss is directly related to throughput, Figure 3 is almost the reverse of the graph for UDP throughput (Figure 2). Since packet loss is due to queue limitations, a higher data rate leads to a lower packet loss and a lower data rate leads to a higher packet loss.

**Figure 4:**
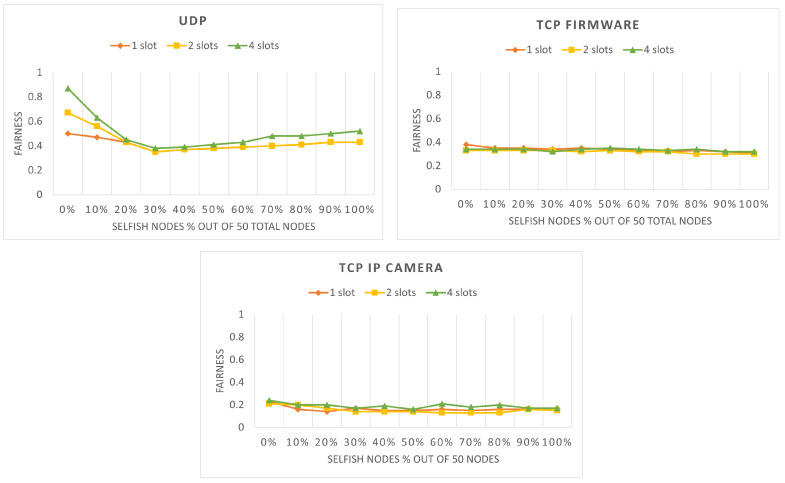
Network fairness with various percentage of selfish stations and RAW slots for 60 s.

The next step of our analysis is to introduce different numbers of selfish stations to study their effects on the network fairness. Figure 4 shows the network fairness for different percentages of selfish stations. As it can be seen, the fairness with any percentage of selfish stations is always lower than the fairness with no selfish stations. Furthermore, in UDP, the number of RAW slots does not play a significant role. Another interesting observation is that, for UDP and TCP IP camera with 30% selfish stations, the fairness is the lowest compared to the cases with higher percentages of selfish stations. The reason behind this is that when the selfish stations are not dominating, they only compete with the honest stations for the channel access, but when the share of the selfish stations increases, they begin to compete with each other. A conclusion that can be drawn here is that compared to TCP, UDP suffers more from the presence of selfish stations in the network. This can be explained by the different nature of the protocols and the lower speed and contention in TCP.

**Figure 5:**
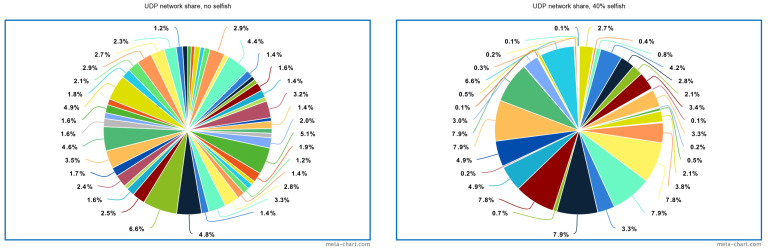
Network share of received packets at the AP for 50 stations divided in two RAW slots for 60 s.

To better illustrate why fairness of the network decreases when more selfish stations are added to the network, the share of the channel access is visualized in Figure 5, which shows the share of packets received at the AP from 50 stations. In a perfect scenario (i.e., 100% fair network) the share of each station must be 2%. As it can be seen, when no selfish stations are introduced, the share is relatively similar, although it is still not the expected 2%. The smallest value is 1.2% and the largest value is 6.6%. This shows that even without introducing selfish stations, the IEEE 802.11ah network is not entirely fair. However, in the case of having 40% selfish stations, the network share changes noticeably. The network share of some stations drops to only 0.1%, whereas others increase to 7.9%. It can be clearly seen that some stations are more dominant than others.

**Figure 6:**
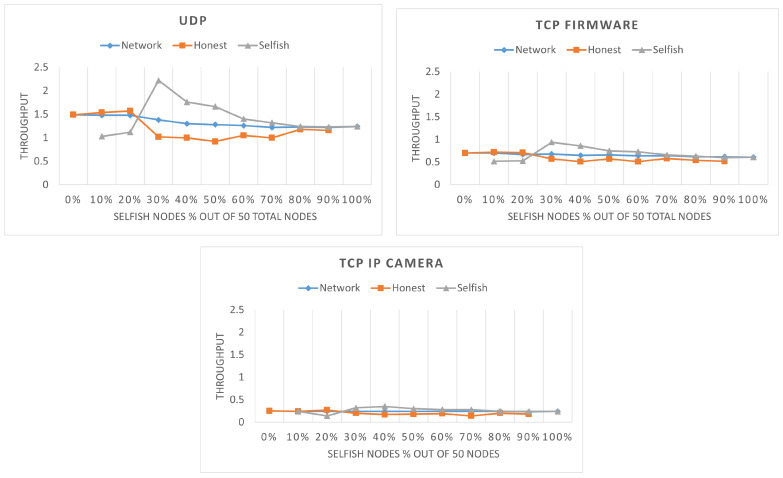
Network throughput (in Mbps) of the network, selfish, and honest stations in 60 s for different percentages of selfish stations in a network with two RAW slots.

So far, we have seen how fairness and network share change with the introduction of selfish stations. Next, it is important to analyze the network quality of service in terms of throughput and packet loss of the honest and selfish stations in the presence of selfish station. As it can be seen in Figure 6, when the selfish stations are fewer than 20% of the network, the throughput of the selfish stations is lower than the throughput of the honest stations. This means that selfish stations do not gain any advantage if they are not more than 20% of network’s stations. The reason behind this is the inherent unfairness of IEEE 802.11ah, which we have shown in Figure 2. For the selfish stations to gain advantages, they must not be selfish individually but as a group (i.e., together with other selfish stations). Looking at Figure 6, 30% of the stations should be selfish to gain significant advantage. In all three scenarios, when selfish stations are 30%, they have the highest throughput, but the advantage is reduced as their number increases further. Finally, the overall network throughput is slightly decreasing for all scenarios, due to the lowered throughput of honest stations.

**Figure 7:**
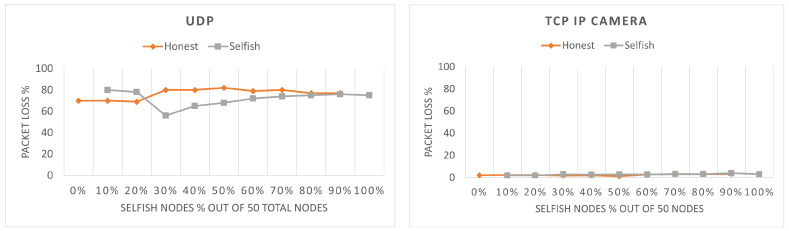
Packet loss of selfish and honest stations in 60 s for different percentages of selfish stations in a network with two RAW slots.

The packets loss can be found in Figure 7. Note that for the TCP firmware scenario, no packet loss was observed; therefore, it is not visualized. As it can be seen, the packet loss is higher for UDP than TCP IP camera, because the packet generation period of UDP is lower than TCP IP camera due to the higher data rate of UDP. Moreover, the honest stations have higher packet loss, because they have a smaller possibility to transmit their packets and, hence, their queue fills faster and empties slower. For TCP IP camera, selfish stations have higher packet loss; however, both selfish and honest stations have relatively low packet loss and the difference between the two is not significant (i.e., 1–2%). The values of the packet loss are dependent on the throughput. A higher throughput will result in a lower packet loss and vice versa. Therefore, packet loss figures are the reverse of throughput figures. This is most clearly noticed for UDP with 30% selfish stations. As it can be seen, the throughput of the selfish stations is lower than the honest stations up to 30% and they also have higher packet loss. At the point of 30%, selfish stations gain higher throughput and their packet loss drops below the packet loss of honest stations.

## 5. Detection of Selfish Behavior

In this section, we analyze two selfish behavior detection algorithms from the literature. After analyzing their applicability to IEEE 802.11ah, we propose a new detection algorithm tailored to IEEE 802.11ah. Through our extensive evaluations with these three algorithms, we determine which one fits the IEEE 802.11ah best.

### 5.1. Principal-Agent Method

The principal-agent method [7] was proposed for IEEE 802.11 wireless ad hoc networks. Due to lack of a central station such as an AP in ad hoc networks, the authors introduced a so called principal station to the network to identify whether a stations is selfish. All other non-principal stations in the network are referred to as agents. The communication in the network works as follows: (i) a transmitting agent sends a RTS to the receiving agent; (ii) the receiving agent sends a query to the Principal to ask whether the sender is selfish; (iii) the Principal computes the utility function (described later) and sends the outcome to the receiving agent; (iv) if the Principal determines that the sender is selfish, the receiver does not respond to the sender; (v) if the Principal determines that the sender is honest, the receiver replies with CTS.

One should note that there are differences between ad hoc networks and star based networks such as IEEE 802.11ah. Firstly, in IEEE 802.11ah, RTS and CTS are disabled to reduce the communication overhead. Secondly, in IEEE 802.11ah there already exists a station that can monitor the entire network and that is the AP. Therefore, some modifications need to be made to apply the algorithm to IEEE 802.11ah. The first and the most obvious one is combining the receiving station and the principal station because in case of IEEE 802.11ah, they both are AP. The second required change is that all packets are received from a station, even if it is detected as selfish, due to the lack of RTS and CTS packets. Modified communication appears as follows: (i) a transmitting agent sends a packet to the AP, (ii) the AP computes the utility function, and (iii) if the AP determines that the sender is selfish, AP marks the sender as such.

The utility function of the principal-agent method is a function that determines whether a station is selfish. If the outcome of the utilization function is less than zero, then the station is selfish and it will spend less time being idle, which results in higher utilization. For the fairness function, the authors used Jain’s fairness function [35].

### 5.2. Deviation Detection Mechanism

The second detection algorithm studied in this paper is the distributed deviation detection mechanism [8], which again is originally proposed for adhoc networks and we need to modify it to work for IEEE 802.11ah. In the original version, every station in the network calculates the mean and standard deviation of the number of packets sent by all stations. Then, whenever a station sends a RTS to a receiver, all stations check the number of total packets sent by the sender. If the total packets sent are greater than the sum of the mean and standard deviation, then the station is considered to be selfish and the receiving station will not respond with CTS.

Our modification of deviation detection from adhoc networks to be applicable to IEEE 802.11ah is straightforward. Instead of all stations computing the statistics of all other stations, only the AP performs this. Furthermore, again because RTS and CTS are disabled in IEEE 802.11ah, the station will only be marked as selfish so that the mitigation algorithm described later in Section 6 can handle it. The drawback of this mechanism is that in cases where selfish stations are dominating the network, the values of the mean and standard deviation are going to be primarily based on the selfish stations and only extreme cases of selfish behavior can be detected.

### 5.3. Inherent Selfish Stations Detector (ISSD)

In this section, we present our new selfish behavior detection algorithm, called Inherent selfish stations detector (ISSD), tailored specifically towards IEEE 802.11ah. We have designed this algorithm considering the fact that IEEE 802.11ah is unfair by design, as we have illustrated in Figure 2. Therefore, we believe that a selfish behavior detection algorithm for IEEE 802.11ah should not only be able to detect the introduced selfish stations but also the inherent selfish stations. This can be achieved by studying the distribution of the network share without the presence of selfish stations.

**Figure 8:**
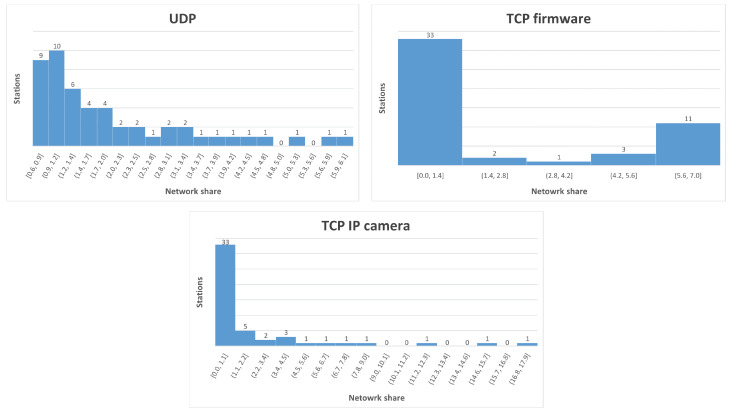
Network share distribution in 60 s for 50 stations divided into two RAW slots.

As it can be seen from Figure 8, even without the presence of selfish stations, most of the stations fall in the lower end of the network share and a small number of stations is having a greater share. Based on this distribution, a threshold value can be introduced for each of the three applications that will detect stations that are behaving as selfish, even though they are not modified to be so. The thresholds have such values that only a few stations that have the greater share of the network will have a greater value. We set for UDP the threshold as 3.1, TCP firmware as 3.34 and TCP IP camera as 3.5. The formal definition of an inherent selfish station, which is also the function used for detection of selfish stations by the algorithm, is station_share > threshold.

The threshold is selected based on the application of the network. Looking at the distributions, UDP has 12 selfish stations, TCP firmware has 15 and TCP IP camera has 7. Therefore, a selfish stations is now a station that has been introduced as selfish or is inherently selfish. Note that the distributions are the average of 10 simulation rounds. When applying the algorithm in the simulations, the number of inherent selfish stations may vary based on the random seed used in the simulation.

### 5.4. Performance Evaluation

To evaluate and compare the previous three detection algorithms, we perform the simulations to be described in this section. The simulation parameters are the same as those described in Section 3. The three detection algorithms described were evaluated for a network of 50 stations and 2 RAW slots. Furthermore, they were also tested for the three different applications: UDP, TCP firmware and TCP IP camera.

#### 5.4.1. Principal-Agent Method

The results of the experiments with the principal-agent method can be found in Table 2, Table 3 and Table 4. As it can be seen, even though all selfish stations are detected successfully, the method detects almost all of the honest stations in the network as selfish as well. This is a very high false-positive rate, which makes the method unusable for IEEE 802.11ah. One of our observations that explains the results is that the average network fairness used in fairness function of this method (reader is referred to [7] for more details about the function) is almost always greater than the network fairness. Furthermore, the current idle time and the average idle time have very small difference and compared to the total transmission time they are insignificant. Therefore the outcome of their utility function is very small and does not play a role in the final outcome because it is always smaller than the difference between the fairness functions. However, the simulated traffic in our study is very intense; hence, the network has very high utilization. The high utilization causes the utilization function to have a very small value compared to the fairness function.

#### 5.4.2. Deviation Detection Mechanism

The results of the experiment with the Deviation detection mechanism can be found in Table 5, Table 6 and Table 7. As it can be seen, the results are better than the ones from the principal-agent method; however, the false-positive and false-negative results are still high. Especially for the case of 20% selfish stations, for UDP and TCP IP camera, only 10% of the selfish stations were detected. However, these results do not mean that the detection algorithm is not working well but rather that the network itself is unfair. As we have illustrated earlier in Figure 5, even without the presence of selfish stations, some stations have as much as five times higher channel share than others. Therefore, it is expected that a good selfish behavior detection algorithm can detect the stations that are inherently selfish. Furthermore, the rate of false negative detected stations increases as the number of selfish stations increases. This behavior is expected, because the selfish stations are dominating the network and being selfish becomes the new norm.

#### 5.4.3. Inherent Selfish Stations Detector

The results of the experiments with our ISSD algorithm can be found in Table 8, Table 9 and Table 10. As it can be seen, the algorithm performs well. When there are no selfish stations, it detects all of the inherent selfish station successfully. Furthermore, because we consider both inherent selfish stations and added selfish stations in detection, the algorithm achieves lower false positive and false negative rates. With the introduction of selfish station, some of the inherent selfish stations may be dominated by the introduced selfish stations. In that case, the inherent selfish stations will no longer be detected as selfish, because they will obtain a low network share; thus, the false negative percentage will increase as it happens in the cases with 10% and 20% selfish stations. Furthermore, because the introduced selfish stations are random, some may overlap with the inherent selfish stations. In the tables with the results, the inherent selfish stations are written in parenthesis on the seconds row of the tables. Overlapping selfish stations are extracted from the number of introduced selfish stations. For example, for UDP with 10% selfish stations, there are ten inherent selfish stations, and five introduced ones. However, one of the introduced stations overlaps with inherent stations, so in the column under 10%, we only see a four on the second row.

### 5.5. Discussion and Comparison

**Figure 9:**
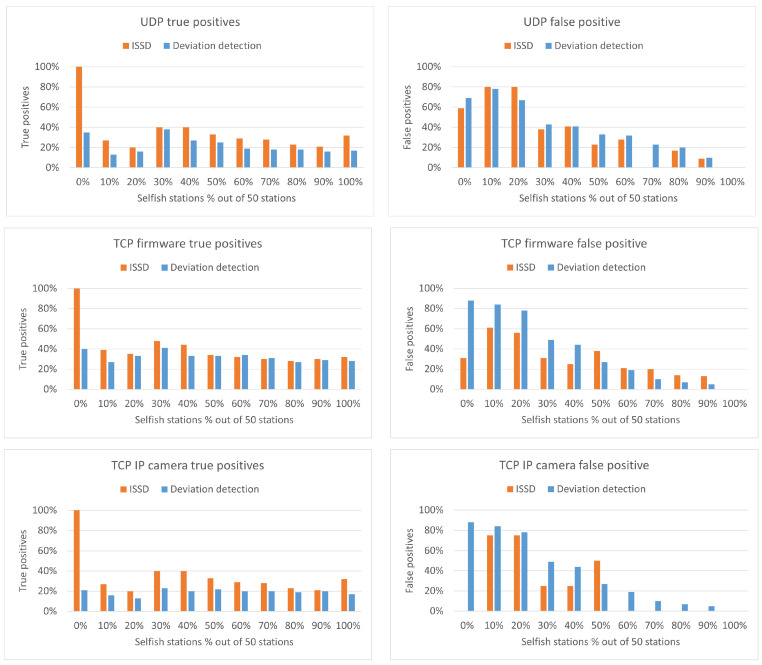
(**left**) True positive. (**right**) False positive—results of the detection algorithms for UDP, TCP firmware and TCP IP camera.

Due to the high false positive and false negative rates of the principle-agent method, we will not continue with the algorithm. Due to different definitions of selfish behavior considered by the deviation detection and ISSD algorithms, we perform a new set of experiments in which not only the introduced selfish stations but also the inherent selfish stations are considered for the results of the deviation detection algorithm. This will allow us to perform a fair comparison between the two detection algorithms. Results of this comparison in terms of true positives can be found in Figure 9 (left). The true positives are calculated by taking the percentage of correct selfish stations detected from both inherent and introduced selfish stations. As it can be seen, ISSD performs significantly better when no additional selfish stations are introduced and it is slightly better for the remaining cases.

A comparison of the false positive rates can be found in Figure 9 (right). As it can be seen, both algorithms follow a similar pattern for the false positive; however, in most cases ISSD has a lower false positive.

**Figure 10:**
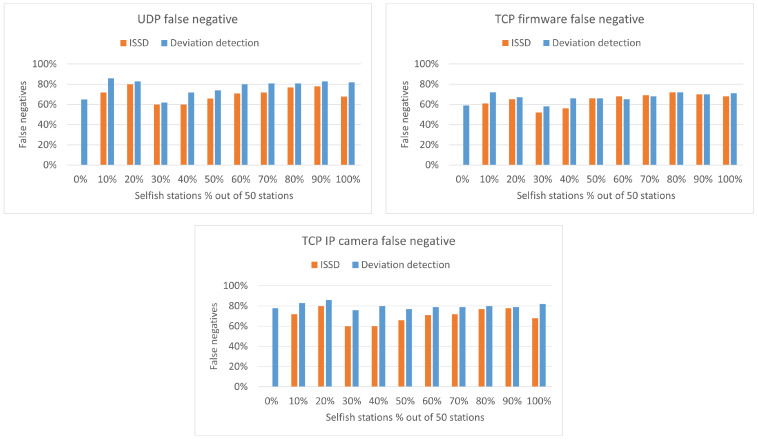
False negative results of the detection algorithms for UDP, TCP firmware and TCP IP camera.

The last metric considered in the comparison is the false negative rate, illustrated in Figure 10. A lower false negative rate means that fewer stations were not detected as selfish although they were. Therefore, the better algorithm is the one with lower value. As it can be seen, ISSD performs better in this respect.

Thus far, we have seen based on the graphs that ISSD performs better compared to the deviation detection algorithm. The last comparison is related to the F1 score, which is calculated using Equation (3). The outcome of the equation varies between 0 and 1 and a higher value means a better score.

(3) $$F_{1}=\frac{TP}{TP+\frac{1}{2}*\left(FP+FN\right)}$$

The F1 scores for both algorithm for the three applications are shown in Table 11. As it can be seen, ISSD has the highest score in all applications. This confirms that indeed, the inherent selfish station detector outperforms deviation detections for the case of IEEE 802.11ah.

The advantages of ISSD combined with the introduction of our “inherent selfish behavior in IEEE 802.11ah” concept allows the following for ISSD: (i) be able to detect selfish stations with higher accuracy, especially when the selfish stations are lower percentages, and (ii) have lower numbers of false positive and false negative outcomes expected due to the refined definition of selfish stations. The drawback of the algorithms is that it is harder to apply it in real scenarios. The traffic of the network is assumed to have similar deterministic pattern, which may not always be the case. Furthermore, the distribution of the network must be obtained first before the threshold can be determined, because the same threshold cannot be used for different applications.

## 6. Mitigating Selfish Behavior

To combat the selfish behavior of selfish stations in IEEE 802.11ah, in this section, we present a new mitigation algorithm called Selfish Stations Quarantine Punishment Algorithm (SSQPA), which enhances the network throughput and mitigates the effect of selfish behavior. We first provide an overview of the design of SSQPA and discuss different variants of it. Subsequently, we present our evaluation results showing the effect of different punishment strategies followed by an analysis on the duration of the punishment. Finally, the breaking point of the number of detected stations will be studied to determine the flexibility of the algorithm.

### 6.1. Selfish Stations Quarantine Punishment Algorithm (SSQPA)

**Figure 11:**
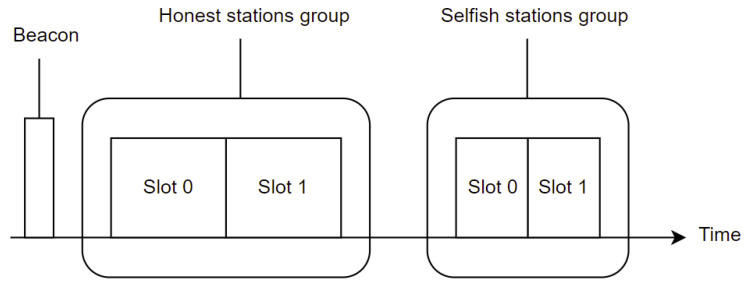
SSQPA RAW grouping.

The SSQPA algorithm aims to optimize the network’s throughput in real time by rewarding stations that obey the binary exponential backoff algorithm of IEEE 802.11ah and punishing stations that are being selfish. The algorithm takes advantage of the RAW allocation of the stations in the network. Once the selfish stations are identified by a selfish behavior detection algorithm, they can be quarantined in a different RAW group from the honest stations for a certain period. IEEE 802.11ah allows dynamic RAW grouping, which means that RAW grouping can be modified with each beacon sent by AP. A visual representation of how SSQPA grouping looks can be found in Figure 11. As it can be seen, honest stations have a RAW group on their own in which they are allocated to different slots. Furthermore, the selfish stations also have a group in which they are placed in different slots. However, the duration of the slots for the group with honest stations will be larger compared to the duration of the slots of the selfish group. In addition, the number of stations allocated to a slot will be smaller for the honest stations. Just like that, honest stations are rewarded by having longer transmission periods for fewer stations and selfish stations are punished, because they will experience higher contention due to the increased number of station per slot and decreased slot duration. There are a few advantages of the proposed grouping compared to the default grouping.

To begin with, honest stations are not experiencing any QoS losses due to the presence of selfish stations in the network. In fact, the honest stations are not even aware of the presence of selfish stations. They will continue to participate in the network normally and will even be rewarded for being honest. Furthermore, selfish stations can continue being selfish but will not experience any gain by doing so. They will only compete with other selfish stations and ultimately will only affect fellow selfish stations. Finally, by keeping selfish stations in the network, it is guaranteed that the packets sent by selfish stations are still received. Even though they are selfish, they might have important data to send to the AP, which must not be lost. It is important to note that a selfish behavior detection algorithm such as the ones presented in Section 2.2 may mistakenly detect honest stations as selfish because they are being awarded while SSQPA grouping takes place. Therefore, to avoid false positive rates, the detection algorithms must not be running while selfish stations are being punished.

Table 12 provides an overview of the parameters used in the algorithm. To begin with, the correct slot format parameter for both groups must be set. For the group of honest stations, the slot format is set to 1, which allows the group to have a total of eigth slots with duration of 246.14 ms. For the group of selfish stations the slot format is set to 0, which means that the slots can be up to 64 but will only be 31.4 ms long. Next, the total number of slots needs to be decided. The two equations used for determining the number of selfish and honest slots per group are Equations (4) and (5), respectively.

(4) $$S_{s}=\min\left(\left\lceil \frac{N_{s}}{50}\right\rceil ,64\right)$$

(5) $$S_{h}=\min\left(\left\lceil \frac{N_{h}}{15}\right\rceil ,8\right)$$

As it can be seen, the allocation is such that honest stations are at most 15 per slot, while selfish stations are at most 50. The values are based on the results of the simulations without selfish stations presented in Figure 2. More specifically, the value for the number of honest stations is drawn based on the results for 30 stations divided into two slots, making 15 stations per slot. As it can be seen, the fairness for that scenario is very high and the honest slots will be aimed at achieving that fairness. One can argue that choosing eight stations per slot may be better because of the higher fairness achieved at the case of 30 stations divided into four slots. However, we did not choose eight as number of stations because it will provided good conditions for the honest stations, as we have seen in Figure 2. The reason for the selfish stations being at a maximum of 50 per slot stems from the case of having 100 stations divided in two slots from Figure 2. As it can be seen, the fairness for this scenario is significantly reduced, meaning that there is high contention between the stations, even without the presence of selfish stations. Since this grouping is rather harsh on the stations, it was decided to be the threshold for the maximal allowed stations per slot for the selfish group. Again, there are scenarios where the fairness is even lower, but these cases will make the punishment too severe and thus are not considered as options. Moreover, if the honest stations are more than 15∗8=120, an additional group for the honest stations is needed. Similarly, if the selfish stations are more than 50∗64=3200, an additional group for selfish stations is required. Finally, the duration of the slots needs to be determined. We define two different variants for determining the slot’s duration, which results in two different variants, i.e., (i) SSQPA–honest stations-centric SSQPA and (ii) network-centric SSQPA. Both variants of the algorithm can be applied to any IEEE 802.11ah network, depending on the goal of the punishment. Algorithm 1 shows the actions needed for the configuration of the RAW grouping discussed above. The following two subsections discuss in greater detail how both variants work.
**Algorithm 1 ** RAW configuration$G_{h}\leftarrow \max\left(N_{h}/120,1\right)$$G_{s}\leftarrow \max\left(N_{s}/3200,1\right)$$S_{h}\leftarrow \min\left(N_{h}/15,8\right)$$S_{s}\leftarrow \min\left(N_{s}/50,64\right)$$NRAWPERBEACON\leftarrow G_{h}+G_{s}$**for ido**    $RawAssignment*m_{raw}\leftarrow newRPS::RawAssignment$    **if** Group is honest **then**        $m_{raw}\to SetSlotFormat\left(1\right)$        $m_{raw}\to SetSlotNum\left(S_{h}\right)$        $m_{raw}\to SetSlotDurationCount\left(NC-SSQPA\left(\right)\right)$    **else if** Group is selfish **then**        $m_{raw}\to SetSlotFormat\left(0\right)$        $m_{raw}\to SetSlotNum\left(S_{s}\right)$        $m_{raw}\to SetSlotDurationCount\left(HSC-SSQPA\left(\right)\right)$    **end if****end for**

#### 6.1.1. Network-Centric SSQPA (NC-SSQPA)

The NC-SSQPA strategy aims to create a fair environment, while at the same time it aims to punish the selfish stations so that they have lower throughput than the honest ones. This is achieved by setting different duration for the slots of both groups based on the number of stations in these slots. As described in Section 1.1, the duration of the slots is determined using Equation (1). Therefore, to select the correct NRAWSlotCount value for each group, i.e, honest and selfish stations, a duration for the slot needs to be selected and the equation needs to be solved for NRAWSlotCount, resulting in Equation (6).

(6) $$NRAWSlotCount=\frac{T_{slot}-500}{120}$$

The duration of the slot is selected based on the number of stations in the group. The two cases for the duration of the selfish slots are shown in Equation (7). In the first case, when the selfish stations are less than 10, $NRAWSlotCount_{s}$ is set to 0, so that the slot obtains a minimum duration of 500 μs after solving the default equation. In the second case, when the station are more than 10, $T_{slot}$ from Equation (6) is chosen such that the duration for the slot is equal to the time needed for stations in the slot to transmit one packet. Therefore, $T_{slot}=P*DR*\frac{N_{s}}{S_{s}}$. As the maximum allowed duration for the slots in the group is 246.14 μs, which is as long as $NRAWSlotCount_{s}<T_{s}$, the minimum between $T_{s}$ and Equation (6) is chosen to follow the specification of the IEEE 802.11ah protocol. If $NRAWSlotCount_{s}>T_{s}$, an error will occur. If the duration of the slot in the first case would have been selected the same way as in the second case, the selfish stations would still experience a fair environment according to Figure 2’s case, with 50 stations with four slots, so that is compensated by significantly reducing slot duration.

(7) $$NRawSlotCount_{s}=\left\{\begin{array}{cc} 0, & \mathrm{if}\;N_{s}\;\leq \;10\\ \min(\frac{P\times DR\times \frac{N_{s}}{S_{s}}-500}{120},T_{s}), & \mathrm{if}\;10\;\leq \;N_{s} \end{array}\right.$$

Next, the duration for the slots of the honest group must be decided. The two cases showing how to choose the appropriate $NRawSlotCount_{h}$ are shown in Equation (8). The first case is when the honest stations are less than 15. Each station will have time to send many packets if the entire remaining beacon interval is allocated to them, so they are restricted to one packet only, similar to the seconds case from Equation (7), so the reward for being honest is slightly reduced. The second case is when the honest stations are more than 15. The total duration of the selfish slots is extracted from the beacon interval and the remaining time is divided equally between all honest slots. In this case as a reward for following the protocol, honest stations are given the remaining of the beacon interval divided by the number of slots. If there is only one slot in the group, it will take the entire beacon interval, and if there are two slots, they will each obtain half of the beacon interval, etc. In both cases, the minimum between the formula and the slot threshold is chosen to prevent illegal values.

(8) $$NRawSlotCount_{h}=\left\{\begin{array}{cc} \min(\frac{P\times DR\times \frac{N_{h}}{S_{h}}-500}{120},T_{h}), & \mathrm{if}\;N_{h}\;\leq \;15\\ \min(\frac{\frac{BI-(Tslot_{s}\times S_{s})}{S_{h}}-500}{120},T_{h}), & \mathrm{otherwise} \end{array}\right.$$

#### 6.1.2. Honest Stations-Centric SSQPA (HSC-SSQPA)

The HSC-SSQPA strategy aims to punish the selfish stations as much as possible and to reward the honest stations as much as possible. This severer punishment is actually achieved by not limiting the award of honest stations and allowing them to experience significant improvement in terms of throughput, especially if they are a small proportion of the network. Compared to the honest stations, the selfish stations are going to experience a very low throughput, with the idea that they can change their behavior and become less selfish.

The duration of the slots is similar to the one of NC-SSQPA. For selfish slots, $NRawSlotCount_{s}$ is chosen according to Equation (9), which is the seconds case of Equation (7). The first case is omitted, because even with a more fair grouping of selfish stations when they are less than 15, honest stations will still have higher throughput. In this case, the punishment will be severer when the number of selfish stations is large enough to reach the threshold of the slot duration as there will be more selfish stations competing for channel access for the same slot duration. For the honest stations, the first case from (8) is omitted, which results in Equation (10). In this variant, as mentioned previously, honest stations are highly favored over selfish stations, because there are no restrictions on the slot duration for honest stations. Therefore, for the honest stations, it will be even better if selfish stations are the majority, because the few honest stations will obtain major time from the beacon interval.

(9) $$NRawSlotCount_{s}=\min\left(\frac{P\times DR\times \frac{N_{s}}{S_{s}}-500}{120},T_{s}\right)$$

(10) $$NRawSlotCount_{h}=\min\left(\frac{\frac{BI-(Tslot_{s}\times S_{s})}{S_{h}}-500}{120},T_{h}\right)$$

### 6.2. Performance Evaluation

Performance evaluation is performed using the simulation parameters described in Section 3. Both variants of SSQPA are evaluated for the three different scenarios: UDP, TCP firmware and TCP IP camera. In all simulations, the networks consists of 50 stations.

#### 6.2.1. Effect of SSQPA

The effect of the punishment using both variants of SSQPA is performed by simulating a network in which selfish stations are punished from the start to the end of the simulation. The simulation period is set to 60 s to match the simulation setup of Section 4. The fairness obtained from the new set of experiments is compared with the fairness of Section 4. Then, the throughput and packet loss of the selfish and the honest stations are compared to evaluate whether the punishment is effective.

**Figure 12:**
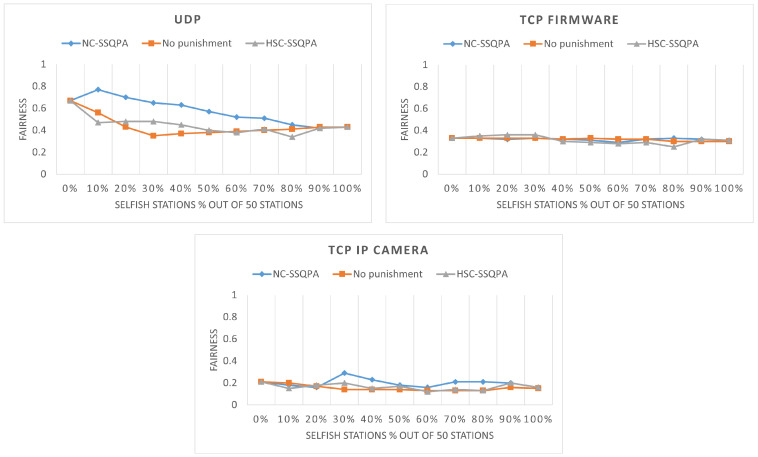
Network fairness comparison between both variants of SSQPA and no punishment.

The fairness comparison can be found in Figure 12. The results are most obvious for the case of UDP. As it can be seen, NC-SSQPA successfully manages to achieve higher fairness compared to both HSC-SSQPA and the case without punishment. In fact, the fairness achieved by NC-SSQPA is similar to the fairness of the case with no selfish stations in the network, i.e, inherent fairness of the IEEE 802.11ah protocol. For TCP IP camera, it can also be observed that NC-SSQPA maintains a higher fairness, although not that significant. Similarly, it can be observed that, for the UDP and TCP IP camera, HSC-SSQPA maintains a level of fairness similar to the one without punishment. Lower fairness for HSC-SSQPA compared to NC-SSQPA is expected due to the nature of both algorithms. The fairness of TCP firmware is not affected by the modified grouping. Judging by the change of fairness alone cannot provide enough evidence to conclude whether selfish behavior is mitigated. Therefore, the throughput of both selfish and honest strategies needs to be compared.

**Figure 13:**
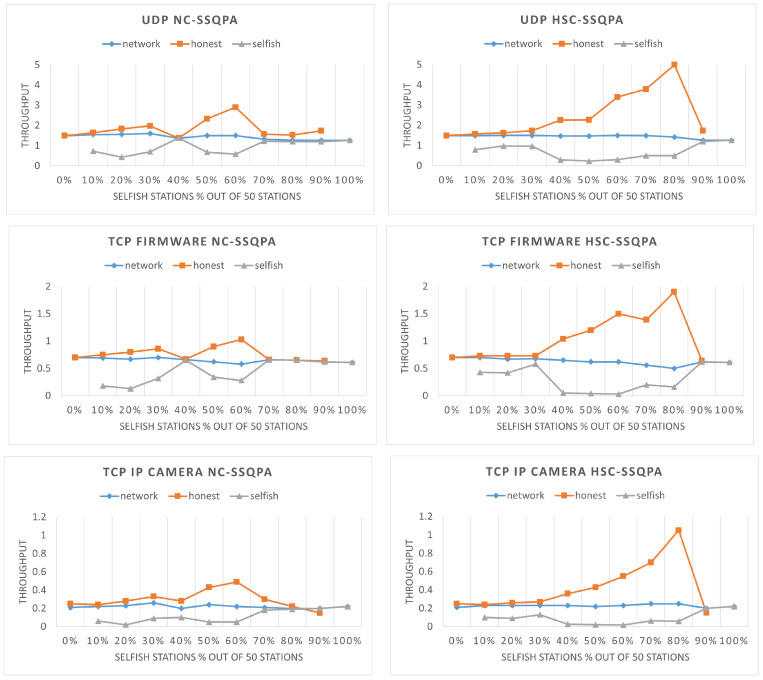
Network throughput (in Mbps) comparison of selfish and honest stations in both variants of SSQPA: (**left**) NC-SSQPA, (**right**) HSC-SSQPA.

Both SSQPA variants manage to successfully mitigate selfish behavior based on throughput comparison. The visual representation of the throughput of the network, selfish, and honest stations can be found in Figure 13. As it can be seen, in all cases, the throughput of the selfish stations is below the throughput of the network. Furthermore, the throughput of the honest stations is above or equal to the overall network throughput. The fact that the line representing the selfish throughput does not proceed above the line representing the honest throughput means that selfish stations do not gain any advantage from being selfish. Thus, selfish behavior is successfully mitigated. Figure 13 also confirms the expectations from the previous section that HSC-SSQPA is more applicable to situations in which honest stations are the minority in the network. This is confirmed by the continuously increasing throughput of honest stations up to an 80% network share of the selfish stations.

The greater the percentage of selfish stations, the greater the throughput of the honest stations. Hence, for selfish stations punished by HSC-SSQPA, it would be best if they are less than 40% of the network. Furthermore, the differences between the throughput of Fair and HSC-SSQPA shows that NC-SSQPA successfully limits the award of honest stations to maintain the fairness of the network. The throughput of selfish stations under NC-SSQPA is reduced at 40% and 70% network share of the selfish stations, which corresponds to the share of selfish stations at which the honest stations are gaining higher throughput under HSC-SSQPA. For selfish stations being punished by NC-SSQPA, it is of their interest to either be 40% or above 70% of the entire network to avoid hard punishment.

Another interesting observation is that regardless of the variant of SSQPA, the overall network throughput remains the same. This, of course, is achieved at the cost of the lower fairness of HSC-SSQPA. In HSC-SSQPA, honest stations have higher throughput compared to the ones of NC-SSQPA, and selfish stations have lower throughput compared to the ones of the NC-SSQPA.

**Figure 14:**
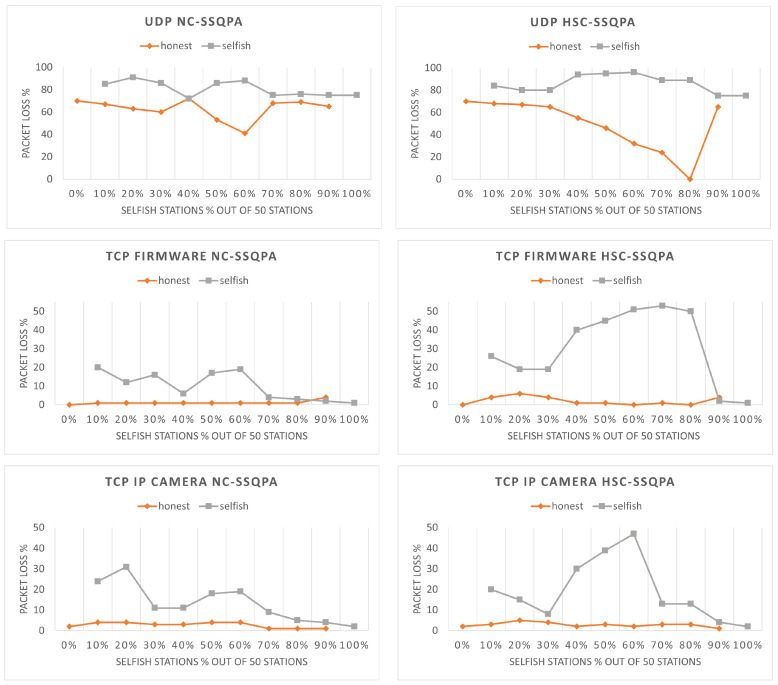
Packet loss comparison of selfish and honest stations in both variants of SSQPA: (**left**) NC-SSQPA, (**right**) HSC-SSQPA.

The final point of performance evaluation of the two algorithms is the packet loss of selfish and honest stations. The results shown in Figure 14 confirm that selfish behavior has been successfully mitigated as, in all cases, selfish stations experience higher packet loss. Higher packet loss is a punishment while low packet loss is a reward. Finally, we observe from Figure 7 that, without a mitigation algorithm, the packet loss of honest stations is higher than the one of selfish stations, which is no longer the case here.

#### 6.2.2. Breaking Point of SSQPA

The breaking point is the threshold for the required minimum percentage of detected selfish stations so that the punishment can still have effects. Knowing this threshold is important because it will indicate whether a detection algorithm is suitable for SSQPA based on its true positive detection rate. After all, SSQPA will not have as good effect, as presented in the previous sections, if the detection algorithm fails to detect the selfish stations. In fact, it may even boost the effect of selfish behavior, because it will allocate selfish stations in slots with lower contention. Therefore, SSQPA must be used only when the detection algorithm meets the threshold requirements.

**Figure 15:**
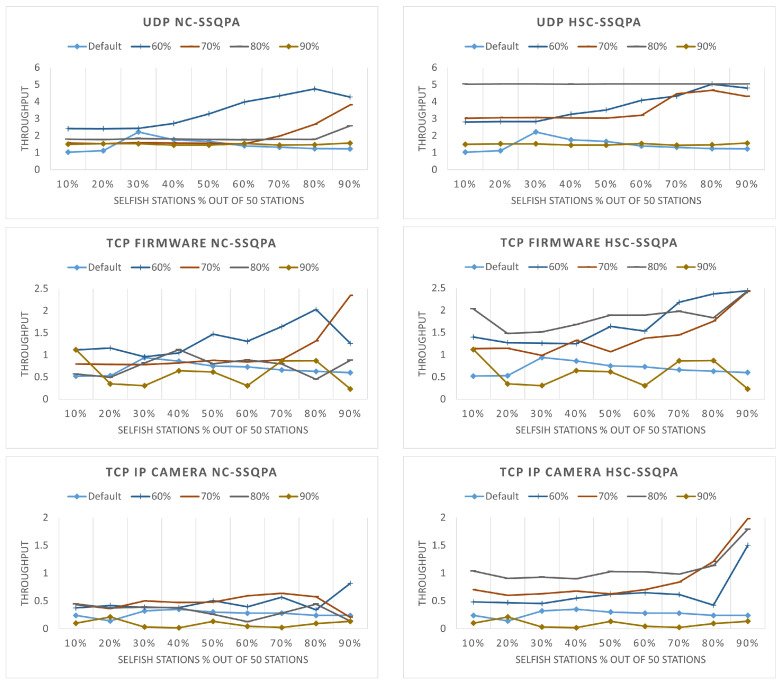
Network throughput (in Mbps) of not punished selfish stations for different percentage of detected stations: (**left**) NC-SSQPA, (**right**) HSC-SSQPA.

The throughput of the selfish stations that have not been punished is illustrated in Figure 15. The default line in the graphs represents the throughput of selfish stations when there is no mitigation technique. An effective punishment is a punishment that does not allow selfish stations to obtain higher throughput than the one they have when there is no punishment. When looking at the graphs of NC-SSQPA, it can be seen that the lines representing 80% and 90% of the detected stations are always below the default one. At 70%, the throughput begins to match the default throughput. When the detected stations drop to only 60%, a significant increase in the throughput can be seen, especially for UDP and TCP firmware. This is not acceptable, because the selfish stations are having higher throughput even than the case when they are not being punished. Therefore, the threshold for NC-SSQPA can be set at 70%, before the selfish stations gain even higher advantage. Similar patterns can be observed for HSC-SSQPA. In all cases, the line of 60% is above all. However, the line for 70% follows the same pattern. Even though in the graphs 70% is below 60%, 70% is still above the default value. Thus, the threshold of HSC-SSQPA is set at 90%.

**Figure 16:**
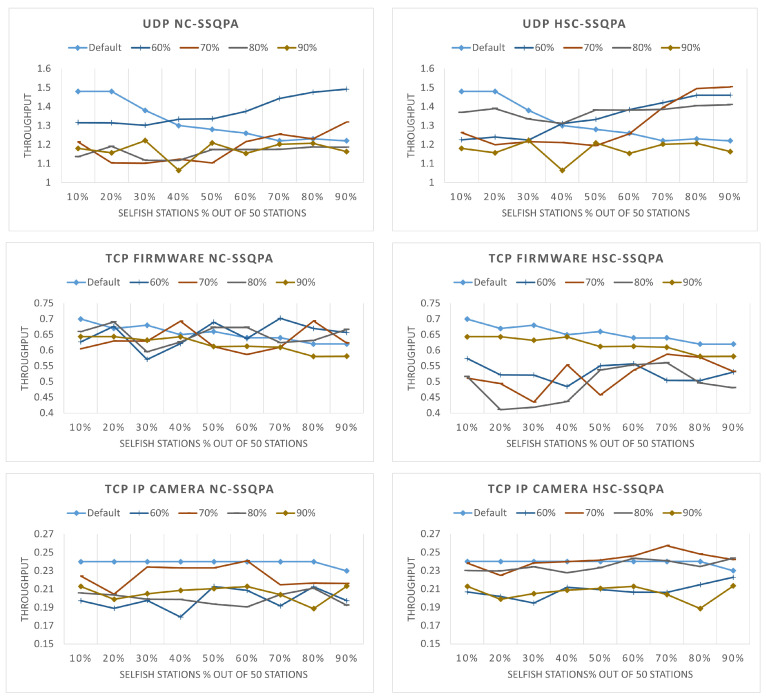
Network throughput (in Mbps) for different percentage of detected stations: (**left**) NC-SSQPA, (**right**) HSC-SSQPA.

We also look into the overall network fairness to analyze how failing to detect the selfish stations will affect QoS. The throughput is illustrated in Figure 16. As it can be seen, for the cases of 90%, 80% and 70%, the throughput is below the throughput for the case without mitigation technique. This shows the negative effect of not being able to detect the selfish stations correctly. The selfish stations that are not being detected (and therefore not punished) dominate the network and as a result, the honest stations are suffering greatly. In the cases where the honest stations are the majority, the overall network fairness is mainly determined by them; thus, fairness is very low. In the cases when the selfish stations are more than the honest, the network fairness is mainly determined by the selfish stations and increases, because being selfish becomes the norm. This is most clear for the cases of 60% detected stations for UDP. Therefore, if the accuracy of the selfish stations detection algorithm is below the identified thresholds, the overall network throughput decreases.

#### 6.2.3. Discussion and Comparison

This section has demonstrated that SSQPA is an efficient countermeasure for selfish behavior. It is now necessary to compare it with other countermeasures to determine whether it is the most applicable for IEEE 802.11ah. The two countermeasures considered are (i) de-association [9] from the network and (ii) packet dropping [9] techniques as these are the most common techniques for Wi-Fi. In the first one, as the name suggests, selfish stations are de-associated from the network and can no longer participate in the communication. This is a harsh punishment that is highly favoring honest stations. In the latter one, selfish stations are not de-associated, but AP drops any packet received from the selfish stations. Essentially, the selfish stations remain in the network and can continue being selfish and take up the communication channel.

**Figure 17:**
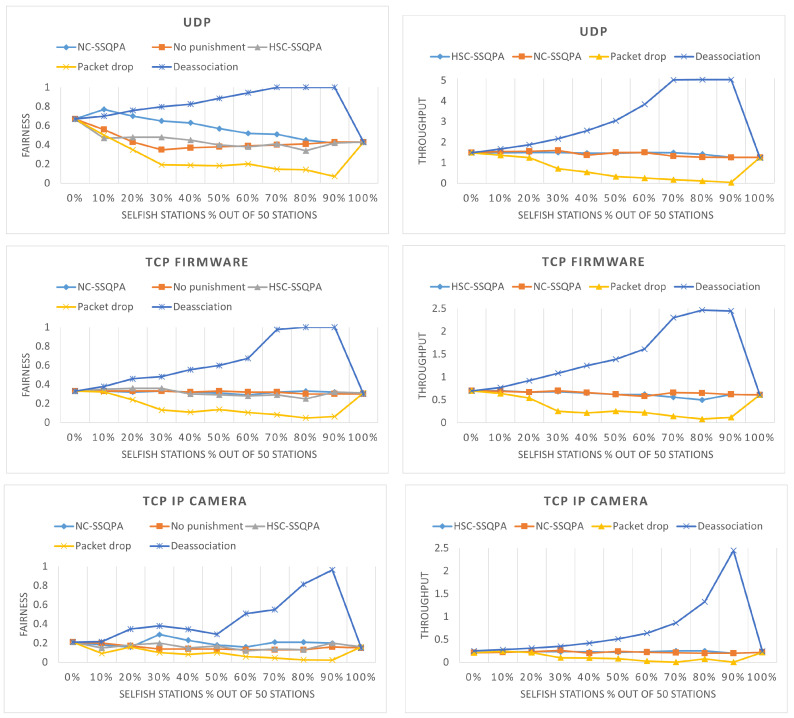
(**left**) Network fairness, (**right**) Network throughput (in Mbps)—for different mitigation techniques.

A comparison between the fairness achieved by both variants of SSQPA, de-association and packet dropping can be found in Figure 17 (left). As it can be seen, packet dropping technique has the lowest fairness, even lower than the case without punishment. On the contrary, de-association achieves the highest fairness, which is even higher than SSQPA. The difference between de-association and packet dropping is so significant because even though the traffic intensity is the same, it is divided over different number of stations. In de-association, the packets sent by the honest stations are divided by the honest stations. In packet dropping, the number of packets sent by the honest stations is divided by the honest and selfish stations. Moreover, de-association achieves much better fairness than SSQPA only when the selfish stations are more than 30%.

Figure 17 (right) illustrates the throughput of the network when the two countermeasure techniques are used, in which a similar pattern as the one of the fairness can be observed. Packet dropping achieves the lowest throughput of all mitigation techniques, while de-association achieves the highest. Again, the throughput of de-association is much higher than SSQPA only when there are more than 30% selfish stations.

Due to the lower throughput and fairness, packet dropping cannot be considered as a good mitigation technique for IEEE 802.11ah. Judging based on the figures only, de-association can be deemed as the best option, because it has better metrics than SSQPA. However, the benefit of using SSQPA is that none of the stations will be disconnected from the network and they will keep transmitting data. Furthermore, the greatest advantage of de-association is when the selfish stations are more than 60%. In that case being selfish is practically the norm of the network and mitigation by de-association will have to de-associate the majority of stations in the network. In that case, SSQPA is better, because instead of allocating all the bandwidth to the minority of honest stations, SSQPA limits the selfish stations and provides better conditions to the honest stations.

## 7. Conclusions

In this paper, we studied the IEEE 802.11ah protocol, also known as WiFi HaLow, introduced by the WiFi Alliance as the WiFi solution for IoT networks.

We first investigated normal behavior of a simulated IEEE 802.11ah network with honest stations only to investigate the default fairness of the network and then gradually introduced different percentages of selfish stations. The simulations included different combinations of RAW slots and various number of stations for UDP, TCP firmware and TCP IP camera. The quality of service parameters in terms of fairness, throughput, and packet loss were analyzed. From our simulation results, it became clear that the IEEE802.11ah network has some inherent unfairness by default. The more stations in the network, the less fair the network became. The highest fairness was achieved when there were 30 stations in the network split into four slots. Therefore, when number of stations increases, fairness can be maintained by increasing the number of slots, because more slots means less stations per slot. After introducing selfish stations, the results showed that the selfish stations need to be selfish together (collaboratively) in order to actually gain advantages. Our results showed that if the selfish stations are less than 30% of the network, they are actually suffering because of the inherent selfish behavior of the network. The selfish stations had the highest advantage when they were 30% to 40% of the network. When they became more than 40%, they began to compete with each other and suffered again. Furthermore, the network’s fairness gradually decreased when selfish stations were introduced. The throughput of selfish stations was higher than the throughput of honest stations when there were more than 30% selfish stations. The reduced throughput of honest stations also led to increased packet loss due to the limited packet buffer size of the stations. Finally, UDP showed to be less influenced by the selfish stations, because it has higher data rates that TCP. Both TCP firmware and TCP IP camera were less fair compared to UDP by default, but the introduction of selfish stations did not affect fairness as significantly as in UDP.

Consequently, we studied applicability of the Principle-agent [7] and Deviation detection [8] for detecting selfish behavior in IEEE802.11ah. Our simulation results showed that the Principle-agent method resulted in extremely high false positive. We expect that it performed poorly because it is designed to work for less traffic-intensive networks. The seconds technique showed better results; however, it still did not perform as expected due to the inherent selfish behavior of IEEE 802.11ah. Therefore, we proposed a new detection algorithm called Inherent Selfish Stations Detector (ISSD) designed to detect not only the introduced selfish stations but also the inherent ones. The proposed technique uses the baseline distribution of the network share to identify a threshold for detection of selfish stations. The comparison with the deviation detection mechanism showed that ISSD performed better for UDP, TCP firmware and TCP IP camera and, thus, may be considered a suitable detection mechanism for IEEE 802.11ah.

Finally, we presented a new selfish behavior mitigation algorithm, called Selfish Stations Quarantine Punishment Algorithm (SSQPA). The algorithm relies on the successful detection of the selfish stations by a selfish stations detection technique. Then, it allocates the identified selfish stations in a separate RAW group dedicated to selfish stations only. By doing so, the honest stations are completely isolated from the selfish ones and no longer suffer from decreased throughput and packet loss. The punishment strategy for the selfish stations is based on reducing their slot duration and increasing the contention. Honest stations were awarded for being honest through increased slot duration and decreased contention. Two different variants of SSQPA were presented: network-centric SSQPA and honest station-centric SSQPA. The goal of NC-SSQPA is to create a fair network, in which the award of honest stations is limited to prevent honest stations from gaining significant advantages. On the other hand, HSC-SSQPA awards the honest stations without any limits in order to create a network that highly favors honest stations but decreases fairness. Our performance evaluation showed that both variants of SSQPA successfully mitigate the negative effect of selfish behavior on the network and can be used for UDP, TCP firmware and TCP IP camera scenarios.

We also studied the breaking point of the algorithm, which is the percentage of correctly detected selfish station required for the SSQPA to be beneficial. The results showed that, for NC-SSQPA, at least 70% of the selfish stations need to be detected, while for HSC-SSQPA, at least 80% need to be detected. Failing to detect that many stations would provide higher gain to the honest stations compared to the case where they are not being punished, because they will be awarded for obeying the protocol, even though they are not obeying the protocol. Therefore, the detection algorithm used for SSQPA must detect at least 70% of the stations for NC-SSQPA and at least 90% for HSC-SSQPA.

The current version of ISSD relies on the manual analysis of network distribution to determine the threshold for detection of selfish stations. This can be improved, in future studies, by implementing an algorithm that calculates the shares and selects a threshold based on the distribution such that the most dominant stations with the highest network share are detected as selfish only. Additionally, the evolution of the population of the network can be studied when all stations can choose their own behavior and the goal of the stations are to maximize their own throughput.

Additionally, in the current version of SSQPA, it is not possible to determine whether a selfish station has changed its behavior. As part of future studies, it would be good to study how introduction of new RAW groups in which selfish stations will be allocated after they have spent some time in the selfish stations group can influence mitigation.

## Figures and Tables

**Table 1:** Simulation parameters.

Parameter	Value	Parameter	Value
CW_min_ honest	15	Max distance to AP	50 m
CW_min_ selfish	2	Simulation time	60 s
CW_max_	1023	Packet size	512 bits
Channel bandwidth	1 Mhz		

**Table 2:** Principal-agent detection method for 50 stations in UDP scenario.

selfish stations %	0%	10%	20%	30%	40%	50%	60%	70%	80%	90%	100%
selfish stations	0	5	10	15	20	25	30	35	40	45	50
correctly detected %	-	100%	100%	87%	100%	96%	97%	89%	98%	96%	98%
false positive	100%	90%	77%	68%	60%	51%	38%	33%	20%	10%	-
false negative	0%	0%	0%	13%	0%	2%	2%	9%	2%	4%	2%

**Table 3:** Principal-agent detection method for 50 stations and 2 slots in TCP firmware scenario.

selfish stations %	0%	10%	20%	30%	40%	50%	60%	70%	80%	90%	100%
selfish stations	0	5	10	15	20	25	30	35	40	45	50
correctly detected %	-	20%	70%	93%	75%	100%	83%	57%	65%	84%	76%
false positive	100%	97%	81%	65%	62%	36%	40%	37%	24%	5%	-
false negative	0%	80%	30%	7%	40%	0%	17%	43%	35%	16%	24%

**Table 4:** Principal-agent detection method for 50 stations and 2 slots in TCP IP camera scenario.

selfish stations %	0%	10%	20%	30%	40%	50%	60%	70%	80%	90%	100%
selfish stations	0	5	10	15	20	25	30	35	40	45	50
correctly detected %	-	40%	50%	60%	75%	60%	53%	51%	45%	42%	48%
false positive	100%	93%	81%	65%	50%	35%	30%	22%	25%	10%	-
false negative	0%	60%	50%	40%	25%	40%	47%	49%	55%	58%	52%

**Table 5:** Deviation detection mechanism for 50 stations in UDP scenario.

selfish stations %	0%	10%	20%	30%	40%	50%	60%	70%	80%	90%	100%
selfish stations	0	5	10	15	20	25	30	35	40	45	50
correctly detected %	-	0%	10%	46%	35%	32%	30%	23%	20%	18%	12%
false positive	100%	100%	90%	50%	46%	28%	25%	0%	12%	0%	-
false negative	0%	100%	90%	53%	65%	68%	70%	74%	80%	82%	80%

**Table 6:** Deviation detection mechanism for 50 stations and 2 slots in TCP firmware scenario.

selfish stations %	0%	10%	20%	30%	40%	50%	60%	70%	80%	90%	100%
selfish stations	0	5	10	15	20	25	30	35	40	45	50
correctly detected%	-	40%	30%	53%	43%	32%	23%	31%	23%	18%	10%
false positive	100%	80%	77%	38%	30%	20%	42%	8%	10%	11%	-
false negative	0%	60%	70%	47%	57%	68%	77%	69%	77%	82%	90%

**Table 7:** Deviation detection mechanism for 50 stations and 2 slots in TCP IP camera scenario.

selfish stations %	0%	10%	20%	30%	40%	50%	60%	70%	80%	90%	100%
selfish stations	0	5	10	15	20	25	30	35	40	45	50
correctly detected %	-	20%	10%	40%	45%	48%	30%	26%	23%	22%	20%
false positive	100%	92%	88%	57%	40%	33%	25%	10%	25%	9%	-
false negative	0%	60%	90%	60%	55%	52%	90%	74%	78%	97%	80%

**Table 8:** ISSD detection for 50 stations with 2 slots in UDP scenario.

selfish stations %	0%	10%	20%	30%	40%	50%	60%	70%	80%	90%	100%
selfish stations (10+)	0	4	8	10	14	19	23	27	30	35	40
correctly detected %	100%	50%	22%	40%	33%	38%	30%	35%	38%	38%	20%
false positive	25%	42%	60%	43%	50%	21%	33%	19%	6%	0%	0%
false negative	0%	50%	78%	60%	67%	62%	70%	70%	63%	62%	80%

**Table 9:** ISSD detection for 50 stations with 2 slots in TCP firmware scenario.

selfish stations%	0%	10%	20%	30%	40%	50%	60%	70%	80%	90%	100%
selfish stations (13+)	0	5	7	10	14	19	21	25	28	31	34
correctly detected %	100%	39%	35%	48%	44%	34%	32%	30%	28%	30%	32%
false positive	31%	61%	56%	31%	25%	38%	21%	20%	14%	13%	0%
false negative	0%	61%	65%	52%	56%	66%	68%	69%	72%	70%	68%

**Table 10:** ISSD detection for 50 stations with 2 slots in TCP IP camera scenario.

selfish stations %	0%	10%	20%	30%	40%	50%	60%	70%	80%	90%	100%
selfish stations (7+)	0	4	8	13	18	23	28	33	36	40	43
correctly detected%	100%	27%	20%	40%	40%	33%	29%	28%	23%	21%	32%
false positive	59%	80%	80%	38%	41%	23%	28%	0%	17%	9%	0%
false negative	0%	72%	80%	60%	60%	66%	71%	72%	77%	78%	68%

**Table 11:** F1 score for deviation detection and ISSD.

	UDP	TCP Firmware	TCP IP Camera
Deviation detection	0.27	0.45	0.24
ISSD	0.48	0.49	0.41

**Table 12:** SSQPA algorithm parameters.

Parameter	Definition	Parameter	Definition
BI	Beacon Interval	$G_{s}$	Number of selfish stations groups
$N_{s}$	Number of selfish stations	$G_{h}$	Number of honest stations groups
$N_{h}$	Number of honest stations	$T_{s}$	Threshold of selfish slot duration
$S_{s}$	Number of selfish slots	$T_{s}$	Threshold of selfish slot duration
$S_{h}$	Number of honest slots	$T_{h}$	Threshold of honest slot duration
$NRawSlotCount_{s}$	Count of selfish slots	*P*	Packet size
$NRawSlotCount_{h}$	Count of honest slots	DR	Data rate

## Data Availability

Data are generated using NS-3 simulator.
